# Deep subwavelength control of valley polarized cathodoluminescence in h-BN/WSe_2_/h-BN heterostructure

**DOI:** 10.1038/s41467-020-20545-x

**Published:** 2021-01-12

**Authors:** Liheng Zheng, Zhixin Liu, Donglin Liu, Xingguo Wang, Yu Li, Meiling Jiang, Feng Lin, Han Zhang, Bo Shen, Xing Zhu, Yongji Gong, Zheyu Fang

**Affiliations:** 1 School of Physics, State Key Lab for Mesoscopic Physics, Academy for Advanced Interdisciplinary Studies, Collaborative Innovation Center of Quantum Matter, and Nano-optoelectronics Frontier Center of Ministry of Education, Peking University, 100871 Beijing, PR China; 2 School of Materials Science and Engineering, Beihang University, 100191 Beijing, PR China

**Keywords:** Two-dimensional materials, Nanophotonics and plasmonics

## Abstract

Valley pseudospin in transition metal dichalcogenides monolayers intrinsically provides additional possibility to control valley carriers, raising a great impact on valleytronics in following years. The spin-valley locking directly contributes to optical selection rules which allow for valley-dependent addressability of excitons by helical optical pumping. As a binary photonic addressable route, manipulation of valley polarization states is indispensable while effective control methods at deep-subwavelength scale are still limited. Here, we report the excitation and control of valley polarization in h-BN/WSe_2_/h-BN and Au nanoantenna hybrid structure by electron beam. Near-field circularly polarized dipole modes can be excited via precise stimulation and generate the valley polarized cathodoluminescence via near-field interaction. Effective manipulation of valley polarization degree can be realized by variation of excitation position. This report provides a near-field excitation methodology of valley polarization, which offers exciting opportunities for deep-subwavelength valleytronics investigation, optoelectronic circuits integration and future quantum information technologies.

## Abstract (web summary)

Here, the authors generate near-field circularly polarized dipole modes in a hBN/WSe_2_/hBN–Au nanoantenna hybrid structure by electron beam excitation, and show nanoscale control of the valley polarization through spatial variation of the electron beam excitation position.

## Introduction

Valley pseudospin, as an intrinsic property related to the extrema in band structure, provides an additional and unique degree of freedom to control behavior of carriers occupied in a specific valley, which is also widely known as valleytronics^1,2^. Valleytronics, as a rising topic in recent years, paves an alternative way for binary information processing and transmission, substantially requiring an inherent property of materials for selective addressing a specific valley via external control. The advent of 2D transition metal dichalcogenides (TMDs) materials, especially monolayer forms, remarkably promotes the development of valleytronics. Monolayer TMDs materials with honeycomb lattice consist of two different kinds of atoms and they possess natural broken inversion symmetry since their two sublattices are occupied. Because of the broken inversion symmetry, the strong spin–orbit interactions split valence bands and the induced broken spin degeneracy in valence bands combined with the time reversal symmetry results in spin–valley inherently coupling which consequently allows for valley-dependent selective excitation by circularly polarized (CP) optical pumping^3,4^. All optical process raised much attention on valley-based researches such as valley polaritons^5–7^, interlayer spin–valley effects^8–11^, valley-selective directional propagation^12–14^, and valley-based topological photonics^15,16^. Besides, the manipulation of valley degree of freedom also plays an imperative part in utilizing spin–valley coupling to develop future quantum science and has been already achieved by metasurface^17^ and chiral Purcell effect^18^.

In TMDs materials, tungsten diselenide (WSe_2_) monolayer is one of van der Waals layered semiconductors with direct band gap, relatively large binding energy, and high carrier mobility. Moreover, WSe_2_ monolayer has stronger spin–orbit coupling (~0.46 eV) compared with MoS_2_ (~0.15 eV)^19^, namely it has potential to possess long-lived valley polarization, demonstrating that WSe_2_ is a prominent material for valleytronics and quantum information transport. Valley-dependent photonics phenomena in WSe_2_ have also been studied, such as spin–layer locking effect^20^, valley coherence^21,22^, and holes valley polarization^23^. Recently, the lightwave valleytronics in WSe_2_ monolayer is reported^24^, showing the lightwave-induced change of the valley pseudospin which provides a method for fast control of electrons at the fundamental quantum level. This work opens the door to systematic valleytronic logic at optical clock rates, and therefore effective manipulation approaches of valley pseudospin at quantum level are urgently needed. However, limited by optical spatial resolution, the deep-subwavelength excitation and manipulation of valley polarization in 2D materials especially in WSe_2_ monolayer are less reported while such investigation is necessary for revealing physical nature of valley polarization as well as prospective compact valleytronic circuits.

Metallic nanostructures represent a versatile platform in modern photonics study that is capable of generating and manipulating electromagnetic field. It has been extensively investigated over the last decade owing to its enhanced local field, prominent control of optical properties and broad spectral responses from ultraviolet to infrared which is dominantly determined by surface plasmon resonance (SPR). Based on these remarkable properties, SPR can effectively realize the control of spontaneous and stimulated radiation of semiconductors through energy transfer process^25–27^. With assistance of metallic nanostructures, WSe_2_ monolayer exhibits potential in field effect transistors^28^, strong coupling^29^, single-photon emission^30^, and dark exciton^31^. Moreover, spatial dimensions of localized surface plasmon resonance (LSPR) induced by optical nanoantenna approach the length scale of atomic/molecular quantum wave functions. Hence, metallic optical nanoantenna may contribute to investigation of valley polarization at deep-subwavelength scale. So far valley polarization phenomena have almost been excited by far-field CP light corresponding to photons with *m* = ±1 states (m represents photon helicity) which satisfies valley-dependent optical selection rules^32^. However, near-field excitation of such effects which may contribute to a deeper investigation of valley polarization has not yet been verified experimentally. Circular dipole mode is associated with those orbital angular momentum-changed transition (*m*_s_ = ±1 states)^33^, and it can be generated in metallic nanostructures as a kind of LSPR, especially in metallic nanoantennas. Therefore, optical nanoantenna with plasmonic circular dipole mode has the possibility to address distinct valleys through near-field interaction with valley materials especially WSe_2_ monolayer, following optical selection rules.

As a noninvasive detection method, cathodoluminescence (CL) microscopy with nanoscale spatial resolution and precise electron impinging position have been successfully used for plasmonic electromagnetic field investigation^34–36^. Under the electron-beam stimulation, localized surface plasmon (LSP) mode of single metallic nanostructure can be effectively excited and the nanoscale move of electron-beam excitation position directly varies the distribution of local density of states (LDOS), contributing to polarized CL signal^37–39^, and directional emission^40^. CL microscopy has also been used to detect emission properties of semiconductors^41–43^. However, compared with bulk semiconductors, it is challenging to measure CL responses of van der Waals layered semiconductors because the creation cross section of their electron–hole pairs are inevitably small. Recently, the h-BN/WSe_2_/h-BN heterostructure has been proved to effectively increase the recombination probability of electron–hole pairs which is benefited from extra electrons and holes in h-BN diffusing into WSe_2_ monolayer and recombining inside, contributing to a great enhancement of CL responses acquired from WSe_2_ monolayer^44^. Hence, CL microscopy has the ability of studying plasmonic nanostructure/WSe_2_ monolayer hybrid structure in a unique way and it is also necessary to explore metal/TMDs coupling mechanism at deep-subwavelength scale especially plasmonic nanoantenna-induced valley polarization and other valley-dependent phenomena with high spatial resolution.

In this letter, we demonstrate that valley polarization can be achieved and controlled in Au nanoantenna and h-BN/WSe_2_/h-BN hybrid structure under electron stimulation with certain excitation positions at deep-subwavelength scale. Under electron-beam impinging, chiral LDOS distribution and CP dipole mode can be excited with impinging positions located at corners of Au nanoantenna. Induced valley polarized CL emitted from hybrid structure which corresponds to A exciton of WSe_2_ monolayer can be measured by CP-resolved CL spectroscopy while there is no chiroptical phenomena can be observed from CL spectra of either constituent part. Valley-dependent optical selective transition is realized by circular dipole (*m*_s_ = ±1) via near-field energy transfer. And the switch of valley polarization states between “turn-on” and “turn-off” can be effectively achieved by altering distributions of electromagnetic mode via moving electron-beam location, leading to selective control of valley polarization at deep-subwavelength scale. The capability of activating valley polarization through electron stimulation provides a characteristic platform to investigate intrinsic mechanisms of valley-dependent phenomena at deep-subwavelength scale and paves the path for valleytronic devices design, quantum circuits integration, and quantum information processing.

## Results

### Characterization of Au nanoantenna and h-BN/WSe_2_/h-BN hybrid structure

**Fig. 1:**
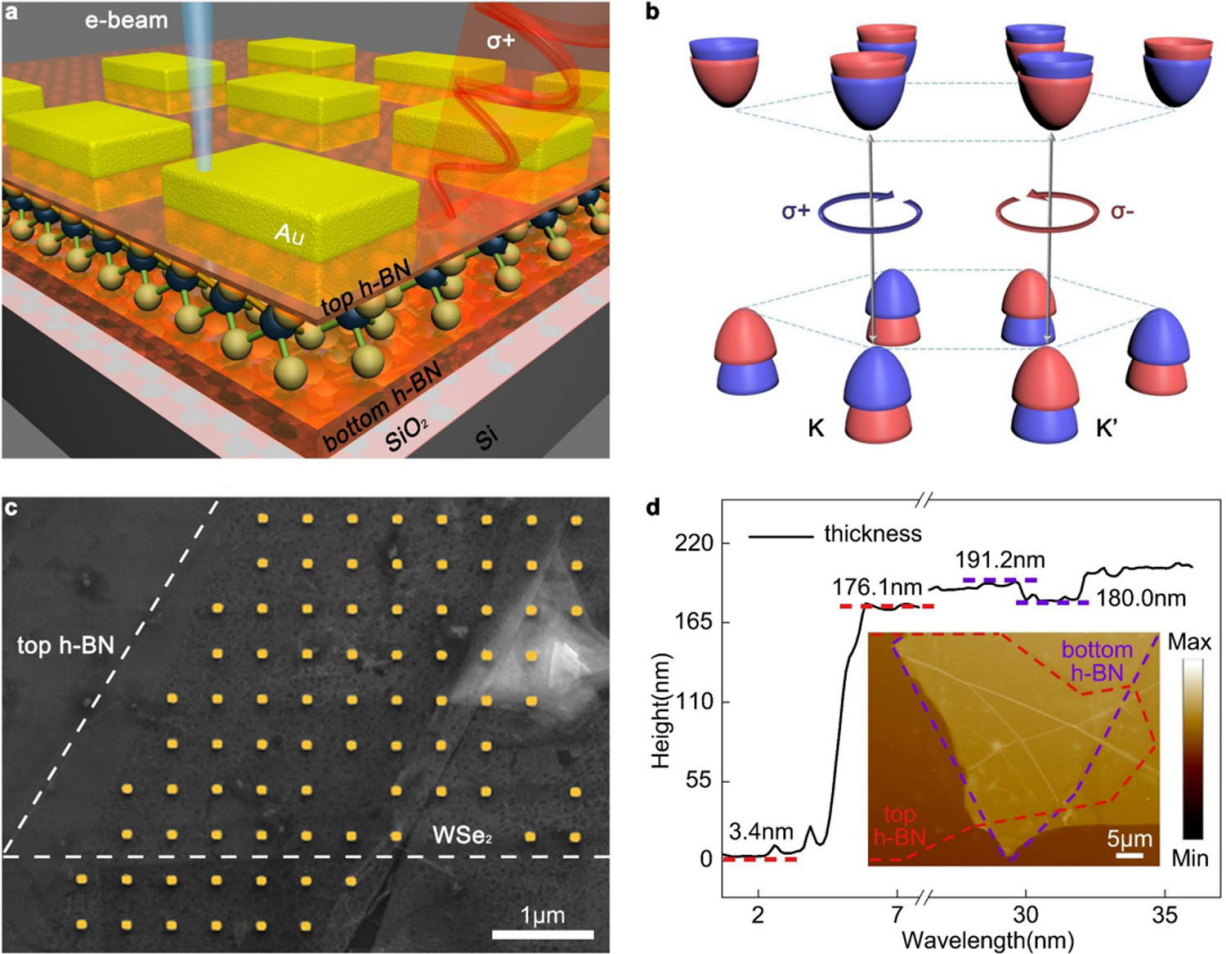
Schematic of the experiment. **a** Schematic illustration of h-BN/WSe_2_/h-BN and Au rectangle nanoantenna hybrid structure, where CVD-grown WSe_2_ monolayer is sandwiched between two h-BN flakes with different thickness under Au nanoantenna, placing over the silicon wafer. Valley polarized CL of WSe_2_ monolayer is excited by LSPR of Au nanoantenna via near-field interaction, under electron-beam stimulation. **b** The hexagonal lattice structure of WSe_2_ monolayer, showing the valley-dependent optical selection rules at distinct K and K’ valleys at the edges of Brillouin zone separated in momentum space but energetically degenerate. **c** The pseudo-color SEM image of h-BN/WSe_2_/h-BN and Au rectangle nanoantenna hybrid structure fabricated by electron-beam lithography, scale bar is 1 μm. WSe_2_ monolayer flake beneath top h-BN is in a shape of part of triangle as the dashed white lines shown. The white triangle on the right of image is the nucleus of WSe_2_ monolayer. **d** The Thickness curve of two h-BN flakes, with top h-BN (11.2 nm) and bottom h-BN (172.7 nm). AFM morphology of hybrid structure is shown in inset and two h-BN flakes are surrounded by red and purple dashed lines respectively, the scale bar is 5 μm.

Figure 1a demonstrates the schematic of coupling structure, with an achiral rectangle nanoantenna stimulated by electron beam. The heterostructure of h-BN/WSe_2_/h-BN was prepared via a series of transfer processes (see “Methods”, Supplementary Fig. 1). h-BN flakes were mechanically exfoliated from bulk h-BN and picked up in sequence by van der Waals force. The chemical vapor deposition (CVD)-grown WSe_2_ monolayer, which is sandwiched between layers of h-BN, was separated from silicon wafer and transferred to cover the bottom h-BN using wet transfer technique^45^. After the construction of layered materials heterostructure, Au rectangle nanoantenna with 127 nm length and 105 nm width was fabricated using electron-beam lithography (EBL) with the deposition of 30 nm Au and 2 nm Ti as adhesive layer.

Figure 1b illustrates the energy band structure of WSe_2_ monolayer, with the spin-split conduction and valence band edges at spatially separated but energetically degenerate K and K’ points in momentum space. The spin orientation of carriers occupied in certain valley is inherently locked to the valley index, allowing optical initialization of valley pseudospin in two inequivalent valleys, with carriers addressable by *m* = ±1 photons (*m* represents photon helicity), represented as σ+ and σ−. As shown in Fig. 1b, the bright excitons at K and K’ valleys only possess in-plane circular polarization $({E_x \,\pm\, iE_y})$ with the opposite helicity. Figure 1c shows the scanning electron microscope (SEM) image of the h-BN/WSe_2_/h-BN heterostructure covered by Au rectangle nanoantenna array with the period of 800 nm and the scale bar is 1 μm. The electron-beam functions as a virtual dipole source enabling LSP mode excitation and manipulation which can be regarded as a point-stimulation method, ignoring the coupling effect between adjacent nanostructures. The vacancy of nanoantenna array obviously shown in SEM picture derives from local fluctuation of h-BN thickness and the relatively thicker heterostructure compared with monolayer material hinders the focusing effect during EBL process which increases the difficulty of fabrication. WSe_2_ monolayer is surrounded by dashed line and the white triangle at top right corner of SEM picture is the nucleus of WSe_2_ that produced during CVD growth process. Au rectangle nanoantenna array can be clearly observed over the top h-BN, appearing on places with and without WSe_2_ monolayer. Two top h-BN with different thickness are transferred onto WSe_2_ monolayer and the gap in the middle of picture distinctly shows the boundary. Figure 1d is the height curve acquired by the atomic force microscope (AFM), showing the thickness of top h-BN (11.2 nm) and bottom h-BN (172.7 nm), with the contractible morphology picture of the sample as shown in inset.

**Fig. 2:**
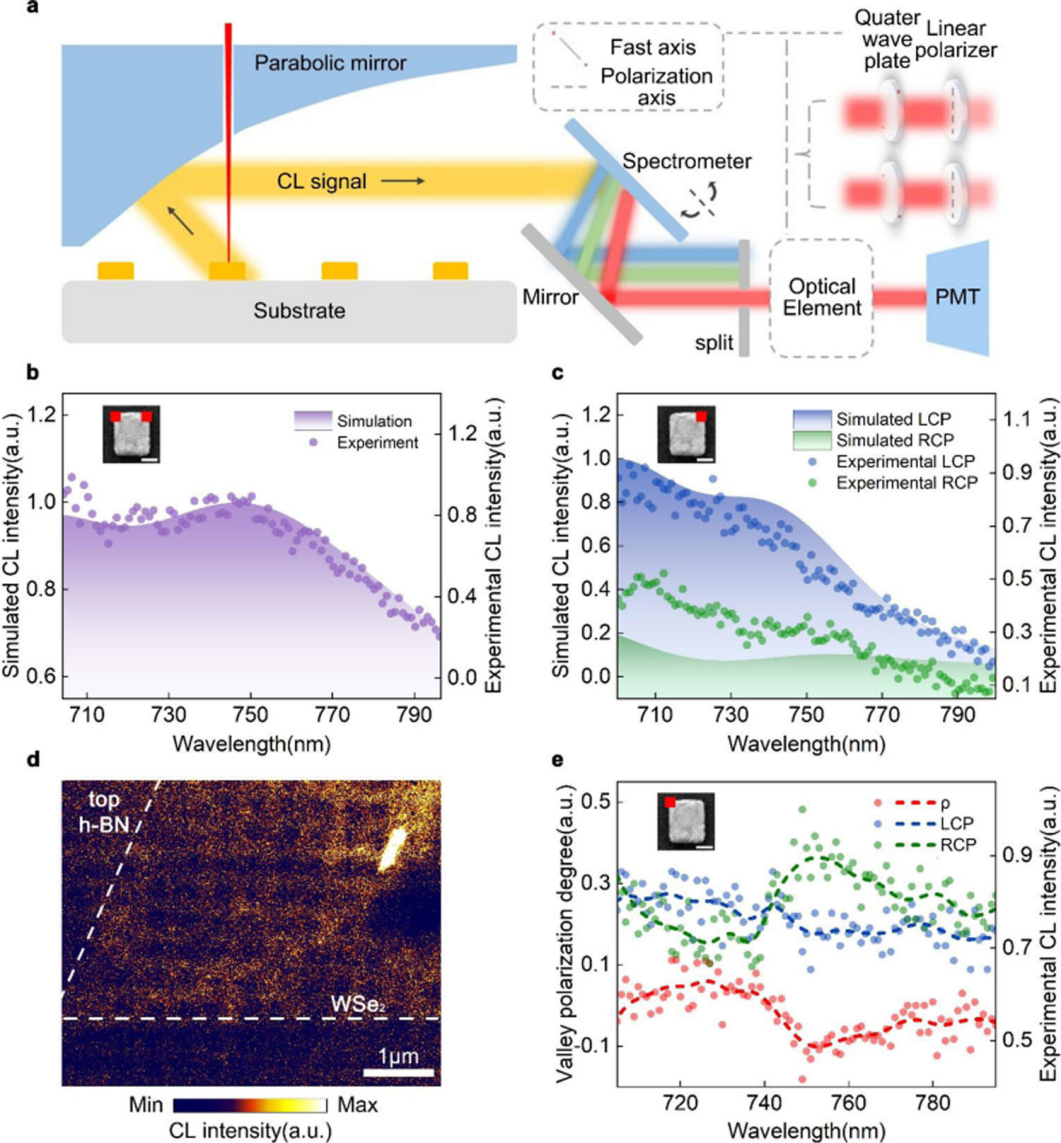
Characterization of the sample and excitation of the valley polarization. **a** The optical path of our CL microscope system for panchromatic CL collection. The quarter-wave plate and linear polarizer are used to extract circularly polarized components of CL emission. **b** Simulated and experimental CL spectra of single Au nanoantenna. The total CL spectra with electron-beam stimulated at top left corner of rectangle shown in inset is the same with that stimulated at top right corner, the scale bar of inset in **b**, **c**, and **e** is 50 nm. **c** Simulated and experimental circularly polarized components of CL emission excited by electron beam located at top right corner. Blue area and blue dots represent simulated and experimental LCP CL spectra respectively, and corresponding RCP components are represented by green area and green dots respectively. Experimental results in (**b**, **c**) were collected under 30 kV electron acceleration voltage, beam current 2.3 nA. **d** The CL mapping image of h-BN/WSe_2_/h-BN heterostructure corresponding to the area in Fig. 1c, and the white shining area derives from the impurity, the scale bar is 1 μm. **e** Experimental LCP and RCP CL spectra obtained from Au nanoantenna/layered materials hybrid structure under 5 keV electron-beam excitation. The stimulation position locates at top left corner of Au rectangle nanoantenna, with the measured valley polarization degree indicated as ρ corresponding to the left ordinate. The dots are experimental results and the dashed lines describe the trend. A linear polarizer (550–1500 nm) and a quarter-wave plate (690–1200 nm) are used to obtain helical components of emitted CL. Experimental results in d and e were collected by 5 kV excitation voltage, beam current 0.6 nA.

All CL measurements were proceeded in a scanning electron microscope (SEM, FEI Quanta 450 FEG) equipped with a specialized CL detection system (Gatan MonoCL4 Plus) (Fig. 2a) which consists of a parabolic mirror, a collection light path and a highly sensitive photomultiplier (PMT). Electron beam passing through the pinhole of the parabolic mirror effectively excited the sample and the CL emission were collected by the mirror and finally acquired at the PMT. A quarter-wave plate combined with a linear polarizer was utilized and placed before the PMT in light path for extracting the LCP and RCP components of CL emissions, respectively. Corresponding CP-resolved CL mapping can be acquired by applying bandpass filters with different central wavelengths. To reinforce the interaction between Au nanoantenna and valley excitons of WSe_2_ monolayer, the LSPR of rectangle structure is designed using finite-difference time-domain simulation (FDTD Solution) whose peak is at 746 nm corresponding to A exciton absorption of WSe_2_ monolayer (Fig. 2b). And large CP luminescence from 700 to 750 nm can also strengthen the chiroptical process (Fig. 2c).

The CL responses of bare Au nanoantenna and h-BN/WSe_2_/h-BN heterostructure were first detected independently. CL signals of single Au nanoantenna were measured by using 30 and 5 kV stimulation voltages, respectively. The resonance peak (30 keV stimulation) was located at 750 nm, agreeing well with the simulation result (Fig. 2b). The far-field CL characterization in Fig. 2c also shows that the LCP intensity is obviously stronger than the RCP with the electron-beam excited at top right corner of nanoantenna. The deviation between experimental results and FDTD-simulated spectra (Supplementary Fig. 2), is mainly caused by the proximity effect where finite data extracted regions ineluctable introduce the response of larger corner area of rectangle nanoantenna, leading to the decrease of measured chirality compared with simulation results. Due to the mirror symmetry, the simulated total CL spectra are the same for top left and top right corner excitations with equal but opposite helical CL emissions (Supplementary Fig. 2). When the stimulation voltage switched to 5 kV with beam current of 0.6 nA, it can hardly obtain any valid CL spectrum from the Au nanoantenna because of the comparably weak far-field emitting efficiency, as shown in Supplementary Fig. 3. However, contrast to the far-field CL spectrum, the near-field distribution of Au nanoantenna resonance mode with its CP-resolved components (Supplementary Fig. 4c, d) still can be clearly observed, with a weaker intensity but the same resonance profile compared with the one excited by 30 kV voltage (beam current 2.3 nA) as shown in Supplementary Fig. 4a, b, which verifies the conclusive excitation of Au nanoantenna plasmon resonance under 5 keV stimulation (Supplementary Notes 2).

As a consequence of the comparably stronger destruction on heterostructure caused by 30 keV electron-beam impinging, the CL spectrum of h-BN/WSe_2_/h-BN heterostructure is measured under 5 keV electron-beam stimulation. The peak locates at 751 nm which corresponds to the A exciton of WSe_2_ and a slight redshift of CL response from photoluminescence (PL) spectrum can be observed (Supplementary Fig. 5), verifying the effective contact between each layer compared with the similar result in previous work^44^. Moreover, the intensity of LCP and RCP components are almost equal, demonstrating that h-BN/WSe_2_/h-BN heterostructure shows no valley polarization phenomenon under electron-beam excitation (Supplementary Fig. 6). When combined with Au nanoantennas, the CL spectrum slightly redshifts with resonance peak located at ~760 nm, which derived from the change of dielectric environment.

### Excitation and manipulation of valley polarized cathodoluminescence

Figure 2d shows the CL intensity mapping of the hybrid structure. A distinguish triangle profile dashed in white lines can be seen which corresponds to the WSe_2_ monolayer exhibited in Fig. 1c, showing that CL signals of WSe_2_ monolayer sandwiched by two h-BN flakes can be actually enhanced compared with single WSe_2_ monolayer (Supplementary Fig. 7). The 732 nm bandpass filter was used to observe the LSP mode variation because this wavelength is away from the WSe_2_ CL peak, and close to the antenna plasmon resonance. The rectangle dark regions in the mapping corresponds to positions of Au nanoantenna and the plasmon mode of single metallic particle as shown in Supplementary Fig. 4c, d decreased in the hybrid structure CL mapping. It demonstrates that near-field coupling between metal nanostructures and h-BN/WSe_2_/h-BN hybrid structure happens and the energy of Au nanostructures flows into the underneath heterostructure through near-field energy transfer, rather than the radiative loss. The detailed analysis of this phenomenon is discussed in Supplementary Notes 1. The twinkle area is caused by the impurities, and the CL intensity of the WSe_2_ nucleus is obviously weaker than the other part of WSe_2_ monolayer, shown as a dark triangle shadow. Figure 2e is the CP-resolved CL spectra with electron beam excited at top left corner of rectangle nanoantenna and it shows an obvious chiral feature, demonstrating the excitation of valley polarization. Here, we define the CL polarization as $\rho \,=\, \frac{\mathrm{CL}_{\mathrm{LCP}} \,-\, \mathrm{CL}_{\mathrm{RCP}}}{\mathrm{CL}_{\mathrm{LCP}} \,+\, \mathrm{CL}_{\mathrm{RCP}}}$, a parameter to characterize the degree of valley polarization. A clear difference between LCP and RCP components exists at resonance wavelength, realizing a −10.9% valley polarization degree at room temperature which is similar with PL valley polarization pumped by CP pumping at room temperature^6^. Owing to the thermal perturbation and deviation, the *ρ* shows a part of positive values while it still presents an obvious valley polarization near the resonance wavelength.

**Fig. 3:**
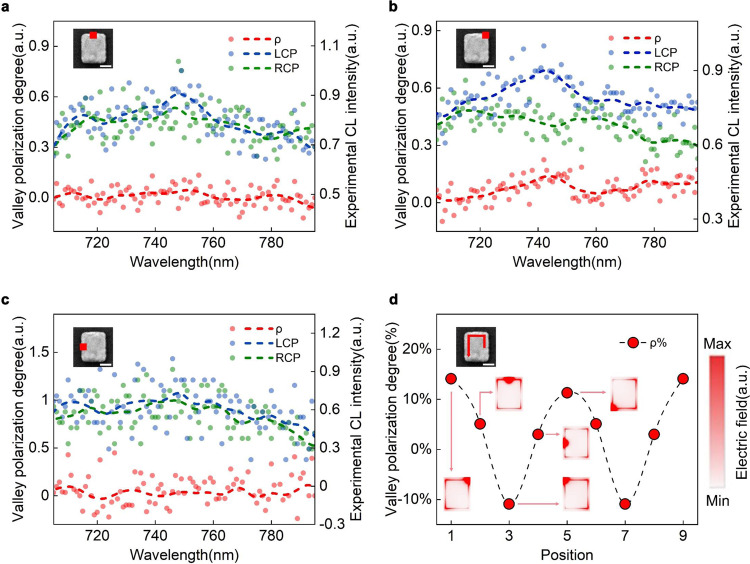
Manipulation of the valley polarization. Normalized LCP and RCP components of CL emitted along +*z* direction obtained by CP-resolved CL microscopy with a linear polarizer (550–1500 nm) and a quarter-wave plate (690–1200 nm) under 5 keV electron-beam excited at (**a**) top middle, (**b**) top right, and (**c**). middle left of nanoantenna with excitation positions shown in insets. The dots are experimental results and the dashed lines describe the trend. The peaks of three spectra almost locate at the same wavelengths with (**a**) 748, (**b**) 742, and (**c**) 747 nm and the fluctuations in spectra may result from thermal perturbation at room temperature and low excitation voltage. **d** Degree of valley polarization calculated at resonance peaks extracted from spectra which were obtained by electron-beam moving along the track represented by red line and arrow shown in inset, and a remarkable ability to control valley polarization is shown as *ρ* changing from almost zero to 14.1% and finally reducing to −10.9% at various excitation positions. The excitation position-dependent simulated electric fields are shown in insets, corresponding to right ordinate.

The ability to manipulate valley polarization degree at deep-subwavelength scale by electron-beam shift is shown in Fig. 3. Four excitation positions are chosen to illustrate the control of valley polarization degree. In Fig. 3a, electron-beam impinging at top middle of nanoantenna results in almost equal CL intensity of LCP and RCP with a valley polarization value as low as about 5% at resonance wavelength, demonstrating a “turn-off” state of valley polarization. However, when the electron-beam moves to top right corner of nanoantenna (Fig. 3b), the reversal CL polarization with LCP intensity obviously larger than RCP component clarifies the sensitive manipulation of valley polarization controlled by electron beam, contributing to *ρ* = 14.1% at resonance wavelength. The calculated *ρ* curve almost over zero shows the regress to “turn-on” state. Finally, shifting to middle left area of nanoantenna (Fig. 3c) returns to “turn-off” state, with *ρ* = 3% at resonance peak and fluctuates around zero. The trivial peak shift of spectra collected among these excitation positions may result from the temperature-induced band gap change of the semiconductor^46^ derived from local heating by electron beam and the waggle of specific stimulation position during measuring which caused by intrinsic astigmatism and perturbance of electron beam at sub-nanoscale. The spectra related to bottom left excitation is shown in Supplementary Fig. 8.

We extract the degree of valley polarization (*ρ*) of these measurements discussed above and illustrate the variation trend in Fig. 3d. With the stimulation position moving along the path shown in inset, it shows a remarkable manipulation ability of CL polarization in this hybrid structure. The switch from “turn-on” state to “turn-off” state can be achieved only within a distance of about 50 nm of the electron-beam shift and the inversion of polarization state can also be realized within about 100 nm (consider the size of stimulation region), demonstrating an efficiently selective control of valley polarization at deep-subwavelength scale accomplished by electron-beam stimulation.

### The theoretical analysis on nanoscale control of valley polarization

Here, we theoretically analyze the intrinsic physics of this intriguing phenomena discussed above. As shown in reciprocity relation between electron beam and plane wave incidence^37^ which derived from Lorentz reciprocity theorem, CP-CL electric field can be straightly associated with CP plane wave incidence induced electric field by the equation:

1 $${\mathbf{E}}_{\rm{CL}}^\sigma ({\mathbf{r}}_0,\,\omega ) \,=\, \frac{{i\omega \rho }}{{4\pi \varepsilon _0c^2R}}e^{ - i\omega t}\int_{ - \infty }^\infty {{\mathbf{E}}_{{\mathrm{PW}}}^\sigma } (x_0,\,y_0,\,z,\,\omega ) \cdot {\mathbf{n}}_{\mathrm{z}}e^{\frac{{ - i\omega z}}{v}}dz.$$

Here, the *ρ*, *ε*_0_, *c*, and *v* are the electron charge, permittivity, light speed in a vacuum, and the velocity of an electron. ${\mathbf{r}} \,=\, (x_0,\,y_0,\,z)$ is the excitation position of the incident electron moving along the −*z* axis, **n**_*z*_ is the unit vector along the +z direction, ${\mathbf{r}}_0 \,=\, (0,\,0,\,R)$ is the far-field CL emission detecting position in a vacuum. From the Eq. (1), the induced electric field ${\mathbf{E}}_{{\mathrm{CL}}}^\sigma ({\mathbf{r}}_0,\,\omega )$ of CL emitted from Au nanoantenna is related with the integral of z component of electric field ${\mathbf{E}}_{{\mathrm{PW}}}^\sigma (x_0,\,y_0,\,z,\,\omega )$ localized in the vicinity of Au nanoantenna generated by normally CP plane wave incidence. The $e^{\frac{{ - i\omega z}}{v}}$ term represents the phase of near-field which directly contributes to chirality of far-field CL emission, making ${\mathbf{E}}_{{\mathrm{CL}}}^\sigma ({\mathbf{r}}_0,\,\omega )$ resolved into circular polarization states ($\sigma \,=\, \pm 1$). Therefore, electron-beam stimulation located at hotspots of chiral LDOS distribution which are induced by CP plane wave incidence, can directly generate CP-CL emission.

As an achiral nanostructure, the rectangle Au nanoantenna with C_2_ symmetry supports two fundamental dipole modes under linear polarized illumination with dipole moment mainly along *x* and *y* axis. And the hybridization of two fundamental modes can be excited by CP plane wave incidence, leading to a chiral electromagnetic field. The 90° phase difference between two fundamental modes combined with extra 90° phase difference inherited from incidence results in chiral plasmonic mode with dipole moment along diagonal line of nanoantenna and the formation of chiral LDOS with hotspots located at top right (top left) and bottom left (bottom right)^39^. The phase difference between two orthogonal fundamental modes makes it responsible to form an in-plane circular dipole mode under electron-beam impinging at hotspots reciprocally. And the mirror symmetry of LDOS distributions and excitation positions demonstrates that the move of electron beam from top right corner to top left corner allows the switch of LCP dipole mode to RCP dipole mode and vice versa.

**Fig. 4:**
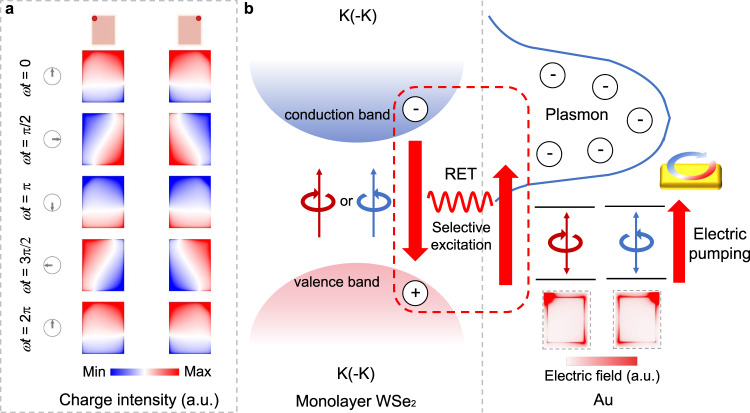
Schematic of the near-field control of valley polarization. **a** Charge distributions of Au rectangle nanoantenna excited by electron-beam impinging at top left and top right corners of rectangle. With helicity-reversed CP dipole transitions, surface charge distributions show counterclockwise and clockwise rotation over time respectively. **b** Schematic of near-field selective excitation process via resonant energy transfer.

The FDTD-simulated charge distributions of top right corner and top left corner excitation are displayed in Fig. 4a. With symmetric excitation position, the corresponding surface charge evolution with time intuitively express the characterization of CP dipole, showing a counterclockwise (clockwise) rotation of the dipole moment. However, the middle left and the top middle excitation cannot generate a CP dipole mode, with dipole moment oscillating along *x* and *y* axis (see in Supplementary Fig. 9). The induced radiation emitted from hotspots of CP dipole mode interfere at far-field region, contributing to CP-CL emission. As one of constituent component of hybrid structure, CP dipole transition $({E_x \,\pm\, iE_y})$ in Au nanoantenna provides the spin angular momentum of light.

In the other way, as another part of constituent component, h-BN/WSe_2_/h-BN heterostructure shows barely valley polarized CL emission under electron-beam stimulation discussed above. Considering the formation of hybrid structure combined with h-BN/WSe_2_/h-BN heterostructure, the incidence and move of electron-beam location directly give rise to the excitation and manipulation of valley polarized CL emission. On account of the exciton diffusion effect^47^, there is no significant influence of the change of electron-beam location on induced emission polarization of WSe_2_ monolayer, which means the non-negligible impact caused by Au nanoantenna. Because the far-field CL radiation emitted from single Au nanoantenna is weak under 5 keV electron stimulation as discussed above, the disturbing effect of far-field responses from Au nanoantenna can be ignored (Supplementary Fig. 10). Therefore, near-field coupling is the dominant interaction mechanism of these deep-subwavelength valley polarization phenomena. Because the top h-BN acts as an insulating layer (10–13 nm average thickness, shown in Supplementary Fig. 11) between Au nanoantenna and WSe_2_ monolayer, the direct electron transfer process can barely happen. The near-field energy transfer must therefore be due to an interaction via local electromagnetic field. The resonant energy transfer (RET)^25^ process is the most probable energy transfer mechanism in this hybrid structure derived from coupling of the large plasmonic dipole moment to the electron–hole pair dipole moment in WSe_2_ monolayer. RET is related with the overlap integral *J* of the interacting dipoles’ spectra as shown in Eq. (2). *F*_Au_ and $F_{\mathrm{WSe}_{2}}$ are the spectra of Au nanoantenna and WSe_2_ monolayer, respectively. As a consequence, the designed almost complete overlap between plasmonic spectrum and CL spectrum of WSe_2_ monolayer can greatly enhance the RET process.

2 $$J \,=\, {\int} {F_{\rm{Au}}} \left( \lambda \right) \,\times\, F_{\rm{WSe}_{2}}\left( \lambda \right) \,\times\, \lambda ^4d\lambda.$$

Furthermore, the change of electromagnetic mode in Au nanoantenna (Fig. 3d) caused by electron-beam moving and corresponding transformation of chiral LDOS distribution consequently give rise to control of valley polarization states, leading to far-field CL polarization detected by CP-resolved CL microscopy. From CP-resolved CL mapping of single Au nanoantenna obtained by bandpass filter with center wavelength located at 732 nm (Supplementary Fig. 4), LCP component of emitted CL corresponds to LDOS hot spots distributing at top right and bottom left corners while RCP component corresponds to those located at top left and bottom right corners of rectangle, agreeing well with analysis results discussed above. When electron-beam impinges at hot spots, two helical-reversed electric dipole modes are generated, exhibiting left-hand (right-hand) circular polarization properties. Because circular dipole corresponds to *m*_s_ = ±1 states, valley-dependent optical selection rules can be satisfied which provides chance for near-field valley polarization excitation. With the move of electron beam, helicity of circular dipole mode is directly altered as well as transforming to linearly polarized dipole mode, followed by tailoring of valley polarization state. Therefore, the localized circular dipole mode of Au nanoantenna directly contributes to the control of valley polarized CL responses and discussions above mainly verify the feasibility of near-filed valley polarization excitation. Figure 4b illustrates the physical mechanism of near-field valley polarization control. CP dipole transitions with helical electromagnetic fields in Au nanoantenna selectively address specific valley excitons through RET, leading to CP emissions. Because excitons of WSe_2_ monolayer and LSP mode are both in-plane circular dipoles, we speculate that it may arise from selective circular dipole–dipole interaction. Furthermore, the Au nanoantenna placed in the vicinity of WSe_2_ monolayer can induce the Purcell effect and lead to a faster recombination rate of valley excitons, which also contributes to the observed valley polarized signals at room temperature. Intriguingly, besides valley polarization, the valley coherence^21,22^ may also be generated by linearly polarized dipole mode, excited by electron-beam impinging at the middle left and the top middle positions of Au nanoantenna, with the dipole oscillated horizontally along the long side and vertically along the short side. The emitted CL signal of WSe_2_ monolayer may be linearly polarized, paralleled to the orientation of linearly polarized excitation.

## Discussion

In summary, we have demonstrated a unique approach for valley-related phenomenon study at deep-subwavelength scale by electron stimulation. Au nanoantenna and h-BN/WSe_2_/h-BN hybrid structure was fabricated and detected by CP-resolved CL microscopy. Plasmonic nanostructure acts as a platform to connect valley carriers in WSe_2_ monolayer with swift impinging electrons, providing an effective method for artificial excitation and manipulation of valley polarization with high spatial resolution. Near-field excitation of valley polarization is verified to be responsible and feasible while the detailed interaction mechanism still needs a deeper investigation. At room temperature, obvious valley polarized CL emission can be acquired and the valley-related spectral variations induced by the change of electron-beam location clarify the sensitive and selective control of such effect. Within about 50 nm distance of electron-beam shift can realize the switch between “on” and “off” states of valley polarization while only 100 nm move can reverse the polarization state. This work enhances the control of valley pseudospin and proposes a powerful route for deep-subwavelength valleytronic researches, opening intriguing avenue for electron–matter interaction and chiral quantum optics. The feasibility to achieve valley-dependent directional propagation^12–14^ in 2D heterostructures with this contractible valley polarized source may provide attractive platform towards miniaturized valleytronic devices.

## Methods

### Heterostructure preparations

First, BN flakes were exfoliated onto polydimethylsiloxane (PDMS) using adhesive tape (USI Adhesive Plastic Film, P/N: 1007R) from synthetic bulk form and suitable h-BN flakes were chosen by optical microscope and AFM, acting as the bottom part of heterostructure with a smooth surface as well as an applicable thickness. And then bottom h-BN was transferred to clean silicon wafer substrate by a precise transfer platform. A CVD-grown WSe_2_ monolayer (Sixcarbon Technology Shenzhen) with triangle shape distributing densely upon silicon wafer was then spin-coated with poly (methyl methacrylate) (PMMA) (Microchem PMMA A7 950) and transferred to cover the bottom h-BN through wet method. The silicon wafer with WSe_2_ monolayer was soaked in concentrated NaOH solution for several hours to corrode silicon dioxide and separate WSe_2_ from substrate. The substrate with bottom BN captures the floating PMMA from beneath and then the silicon wafer was drenched in water to clean the residual NaOH. The sample was 50 °C heated for 5 min to evaporate residual water and PMMA was cleaned away by acetone vapor from 210 °C heated acetone solution. Suitable top h-BN was exfoliated to another silicon wafer substrate examined with the same method as bottom h-BN. Poly-propylene carbonate (PPC) was spun on top of a PDMS which is placed on a glass slide attached by double-sided tape. Top h-BN was picked up by PPC through 50 °C heating and released onto WSe_2_ monolayer to build heterostructure via 100 °C heating by precise transfer platform. The sample was then steeped in chloroform solution for 5–10 min to eliminate the residual PPC and therefore van der Waals’ force can adhere each layer tightly with an effective contact without bubbles.

### CL and AFM measurements

CL signals were obtained by the SEM (FEI Quanta 450 FEG) based CL detector system (Gatan MonoCL4 Plus). The emissions passing through the optical path were acquired by a high-sensitive PMT (HSPMT, 160–930 nm). For CP-resolved CL measurements, the combination of a quarter-wave plate and a linear polarizer was used for wavelength 700–800 nm. The quarter-wave plate and linear polarizer used in experiments were purchased from THORLABS and their modal numbers are AQWP10M-980 and LPVIS100, respectively. Supplementary Figure 12 is the measured CP-CL signals of silicon wafer substrate, which shows the calibration of the polarization response. Locating the fast axis of the quarter-wave plate by ±45° with respect to the polarization axis of linear polarizer can selectively extract LCP and RCP components. CL images with specific wavelength were acquired by applying bandpass filter in the optical path. The thickness of h-BN flakes was measured using a Bruker Dimension Icon AFM in scanayast mode.

### Numerical simulations

All simulation results in this report were all accomplished by commercial finite-difference time-domain methods solver (FDTD Solutions, Lumerical). The simulation domain consisted of structures and perfectly matched layers in *x*, *y*, *z* directions. For calculations of CL emission, electron moving along −*z* axis acting as illumination source was regarded as a linear current density ${\mathbf{J}}({\mathbf{r}},\,t) \,=\, \rho v\delta (z \,+\, vt)\delta (x \,-\, x_0)\delta (y \,-\, y_0){\mathbf{n}}_z$, where *ρ* is the electron charge, *v* is the velocity of electron, ${\mathbf{r}} \,=\, (x_0,\,y_0,\,z)$ is the position of electron beam, and **n**_*z*_ is the unit vector along +*z* directions. In the frequency domain, **J**(**r**, *t*) transforms to ${\mathbf{J}}({\mathbf{r}},\,\omega ) \,=\, \rho e^{\frac{{ - i\omega z}}{v}}\delta (x \,-\, x_0)\delta (y \,-\, y_0){\mathbf{n}}_z$ and electron beam was modeled as a series of dipoles with a phase delay $\frac{z}{v}$. Here, $v \,=\, 0.34c$ (*c* is the speed of light in a vacuum) corresponds to 30 keV electron energy. A reference simulation without any nanostructures and substrate was also run to avoid any background signals generated only by electron beam which could obscure the signals from nanostructures. The far-field CL spectra were calculated by integrating the Poynting vector normal to an arbitrary surface in the upper *z* half-plane for the wavelength from 650 to 800 nm. In sphere coordinates, the time-averaged Poynting vector can be expressed as $P_{{\mathrm{total}}} \,=\, \frac{{\varepsilon _0c\left( {\left| {{\mathbf{E}}_\theta } \right|^2 \,+\, \left| {{\mathbf{E}}_\varphi } \right|^2} \right)}}{2}$, $P_{{\mathrm{LCP}}} \,=\, \frac{{\varepsilon _0c\left( {\left| {{\mathbf{E}}_\theta \,-\, i{\mathbf{E}}_\varphi } \right|^2} \right)}}{4}$, $P_{{\mathrm{RCP}}} \,=\, \frac{{\varepsilon _0c\left( {\left| {{\mathbf{E}}_\theta \,+\, i{\mathbf{E}}_\varphi } \right|^2} \right)}}{4}$ for the total, LCP, RCP CL spectra calculations. The far-field region was set as a vacuum. The materials of nanostructure and substrate used in the simulation were Au (gold) CRC and Si (Silicon)-Palik, respectively, and the refractive index of SiO_2_ was set as 1.5. The minimal mesh was 2 nm and the mesh refinement was chosen as conformal variant 1.

## Supplementary information

Supplementary Information

## Data Availability

The data that support the findings of this study are available in “Science Data Bank”, “http://www.scidb.cn/api/sdb-personal-service/dataset/surl/uY3auq”.
