# Supplementary material for: Deep subwavelength control of valley polarized cathodoluminescence in h-BN/WSe2/h-BN heterostructure
Source: Nat Commun. 2021 Jan 12;12:291. doi: 10.1038/s41467-020-20545-x (PMC7804183; doi:10.1038/s41467-020-20545-x)
Supplement: Supplementary file 1 — Supplementary Information [file 41467_2020_20545_MOESM1_ESM.pdf]

Supplementary Information

Deep subwavelength control of valley polarized cathodoluminescence in h-BN/WSe_2/h-BN heterostructure

Liheng Zheng[1^], Zhixin Liu[1^], Donglin Liu[1], Xingguo Wang[2], Yu Li[1], Meiling Jiang[1], Feng Lin[1], Han Zhang[1], Bo Shen[1], Xing Zhu[1], Yongji Gong[2], Zheyu Fang[1]*

[1]School of Physics, State Key Lab for Mesoscopic Physics, Academy for Advanced Interdisciplinary Studies, Collaborative Innovation Center of Quantum Matter, and no-optoelectronics Frontier Center of Ministry of Education, Peking University, Beijing 100871, China

[2]School of Materials Science and Engineering, Beihang University, Beijing 100191, P. R. China

[*]Email： zhyfang@pku.edu.cn

Supplementary Notes 1: Rectangle dark regions in CL mapping of hybrid structure

In previous reports [*Nat Mater* **3**, 404, 2004. *Nano Lett* **19**, 3543, 2019. *ACS Nano* **13**, 12184, 2019. *etc.*], h-BN, as an insulator with a direct bandgap of 5.971 eV, has been clarified as a well-performed spacer to protect luminescent material from the quenching effect. Besides, in our experiment, different areas of top h-BN (in the vicinity of Au nanoantennas) were scanned by atomic force microscope (AFM) to determine its thickness, as shown in Supplementary Figure 11, where we can see the top h-BN layer shows an excellent homogeneity with the thickness of 10-13 nm, which further confirms the little possibility of luminescence quenching effect via charge transfer. Because the LSP energy is higher than the WSe_2 exciton, thus the main energy transfer direction is from the Au antenna to WSe_2. Meanwhile, the LSP resonance is broad and overlapped with the WSe_2 emission, therefore, the energy transfer from WSe_2 to Au antenna may also exist. However, Fig. 2e and Fig. 3b tell us that there is an obvious valley polarization phenomenon, thus we can conclude that most of the energy transfer is from Au antenna to WSe_2.

In fact, the dark rectangle area in Fig.2d represents the energy transfer process that existed between Au nanostructure and WSe_2 monolayer. The CL mapping of hybrid structure and bare Au nanoantenna that shown in Fig.2d and Supplementary Figure 4c-d were acquired under 5kV excitation voltage by using the same bandpass filter with its center wavelength located at 732nm. The reason we choose this 732 nm wavelength is to clarify the existence of the energy transfer process from the change of plasmon resonance. The CL spectrum of bare Au nanoantenna exhibited in Fig.2b shows a broad resonance, and the CL intensity at 732nm is almost the same as the resonance peak. But in the CL spectrum of hybrid structure (Supplementary Figure 10), the wavelength of 732nm is far away from the resonance peak position (~760 nm), and with much lower intensity compared with the peak. Therefore, the CL mapping at 732nm wavelength can be used to clearly show the plasmon resonance change of the Au nanoantenna with and without h-BN/WSe_2/h-BN heterostructure under less impact of WSe_2 signals. In Supplementary Figure 4c, d, the plasmon mode distributions can be clearly observed

under 5kV electron acceleration voltage. However, in the CL mapping of hybrid structure (Fig.2d), the intensity of plasmon mode distributions decreases, and the position of Au antenna becomes to a dark area with the energy of localized plasmon resonance transferred to the underneath WSe_2 monolayer.

Furthermore, in the experiment, all of the CL measurements of hybrid structure were obtained by using 5kV electron acceleration voltage in order to avoid the destroy of WSe_2 monolayer, and therefore the emission of WSe_2 is really weak. Besides, with the 732nm bandpass filter that used in the CL mapping further decreases the WSe_2 signal (~760 nm) collection efficiency. With the physical shielding of Au nanoantenna on the top of hybrid structure, it finally results in the dark areas as shown in Fig.2d. After we proved the energy transfer from Au nanoantenna to WSe_2 monolayer, we further measured the WSe_2 CL emission spectrum, and valley polarized CL signals that we show in the manuscript as Fig. 2e and Fig.3b clearly clarify the existence of nanoscale manipulation of valley polarization that derived from Au nanoantenna.

Supplementary Notes 2: The influences of excitation voltage and beam current of electron beam to the CL intensity

In our CL measurement for the Au antenna, the scanning electron beam functions as a virtual dipole source. When the swift electron crosses the interface between different dielectrics, the sudden annihilation of its image acts like an induced dipole that produces radiation. [García de Abajo, F. J. Optical excitations in electron microscopy. Rev. Mod. Phys. 82, 209-275 (2010).]

But the coherent dipole radiation yield is directly associated with electron energy. The larger electron acceleration voltage produces the faster electron and the larger beam current produces more electrons to interact with the sample, contributing to the stronger radiation. Therefore, 5 keV, 0.6 nA and 30 keV, 2.3 nA induce the same localized plasmon resonance but with different far-field emission intensity.

Supplementary Figure 1:

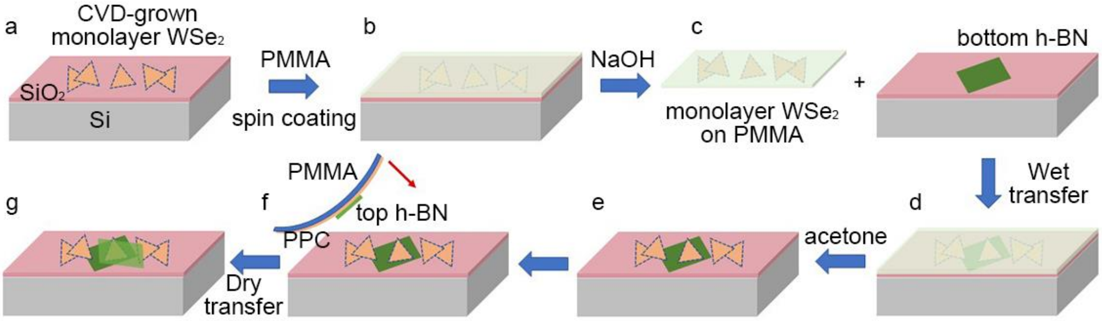

Schematics of wet transfer and dry transfer process to fabricate h-BN/WSe_2/h-BN van der Waals heterostructure.

Supplementary Figure 2:

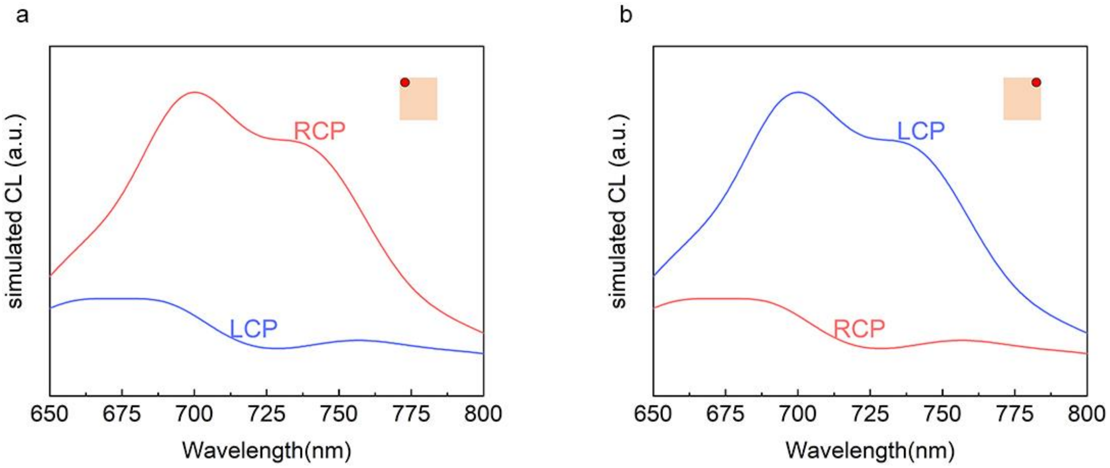

Simulated LCP and RCP components of Au nanoantenna with electron beam excitation position at top left corner and top right corner.

Supplementary Figure 3:

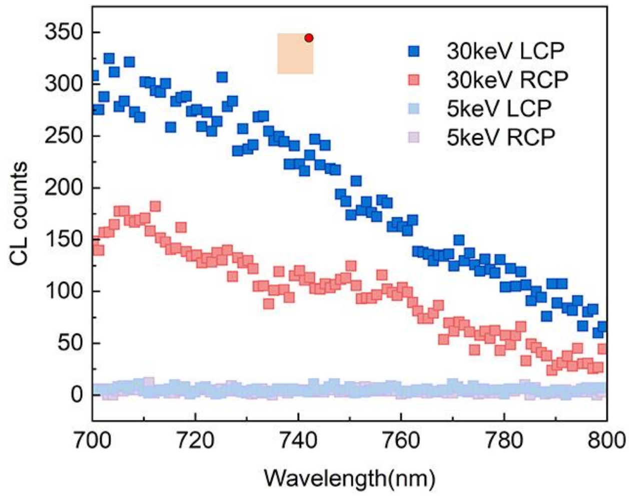

LCP and RCP components of CL emissions from Au nanoantenna excited by electron beam impinging at top right corner of rectangle as shown in inset. Under 30keV, 2.3nA electron beam stimulation, CL signals are strong to be collected and the difference between LCP and RCP components are obvious, resulting in the chiral emission. While under 5keV, 0.6nA electron beam stimulation, few signals can be acquired, showing that there are barely chiroptical responses emitted from Au nanoantenna.

Supplementary Figure 4:

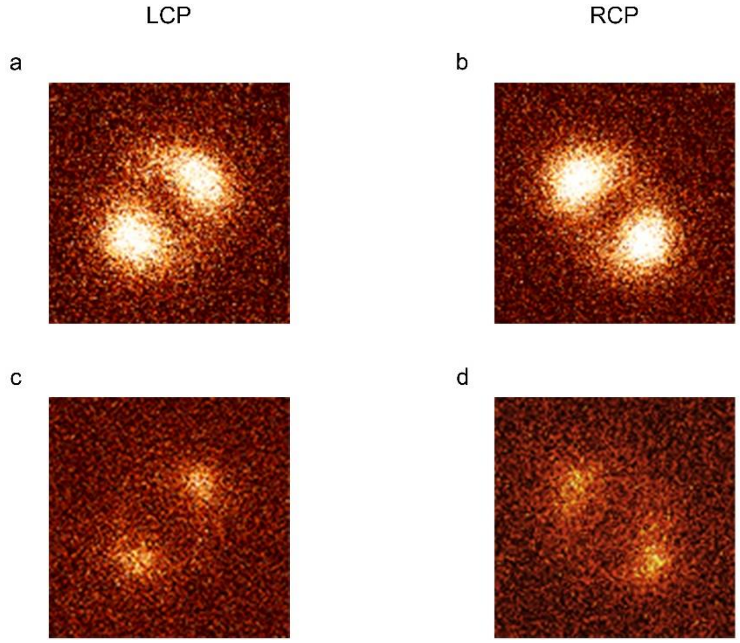

CL mapping of Au nanoantenna acquired by CP-resolved microscopy under a)-b): 30keV, 2.3nA electron beam excitation and c)-d): 5keV, 0.6nA electron beam excitation. The chiral LDOS distributions demonstrate the origin of far-field CP CL emissions and the transformation of LDOS between LCP and RCP components caused by electron beam shift contributes to the inversion of CL CP states. The chiral LDOS distributions show similar plasmon resonance profiles but different CL intensity between 5keV and 30keV stimulation. Therefore, 5keV electron beam stimulation can effectively generate chiral LDOS distributions and the circular electric dipole in Au nanoantenna. This result shows that WSe_2 monolayer can be influenced by near-field LSP mode under 5keV, 0.6nA electron beam excitation.

Supplementary Figure 5:

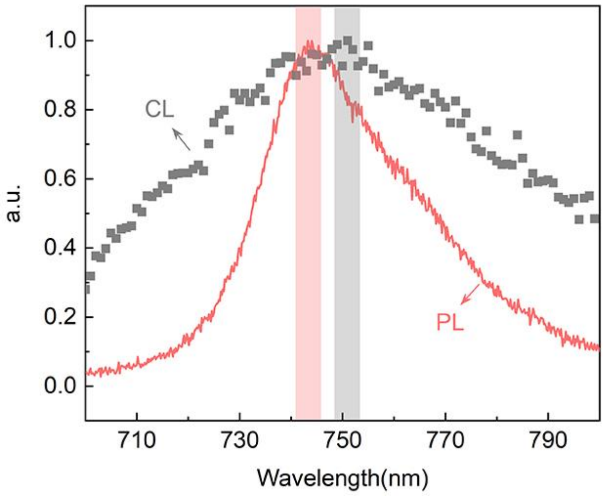

Comparation between CL and PL spectra of h-BN/WSe_2 monolayer/h-BN heterostructure. A small redshift of CL spectrum compared with PL spectrum can be seen which arises from the temperature-induced bandgap shrinkage of semiconductor caused by local heating by electron beam impinging.

Supplementary Figure 6:

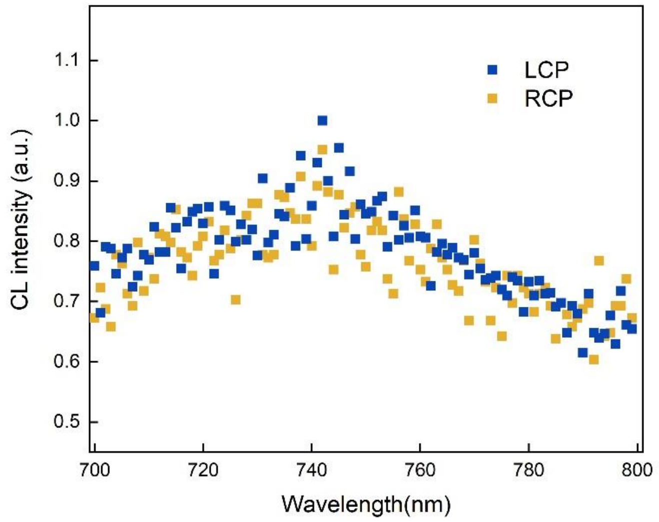

LCP and RCP components of CL emitted from h-BN/WSe_2/h-BN heterostructure obtained by CP-resolved CL spectroscopy under 5keV electron beam excitation. There is barely apparent difference between LCP and RCP emissions without Au nanostructure, showing that electron beam stimulation without spin angular momentum injection cannot generate valley polarization phenomenon in WSe_2 monolayer.

Supplementary Figure 7:

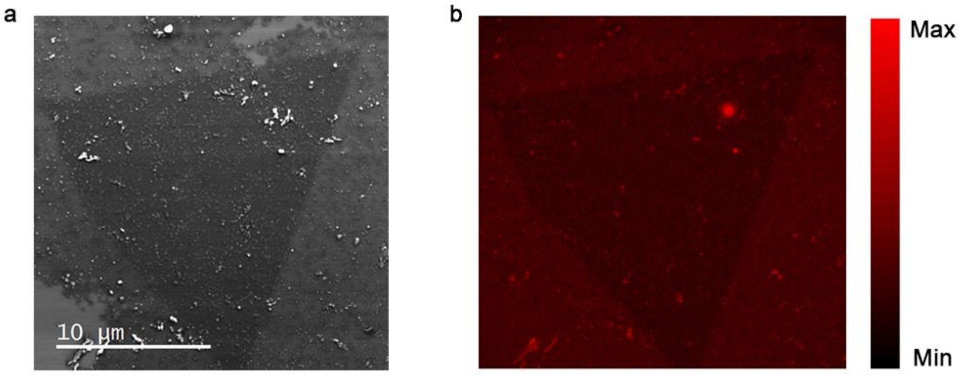

a. the SEM image of a monolayer WSe_2. b. the CL mapping of this WSe_2, and the dark triangle shows that monolayer WSe_2 without h-BN to form a heterostructure cannot produce effective CL signal. The shining area inside the WSe_2 derived from the impurity.

Supplementary Figure 8:

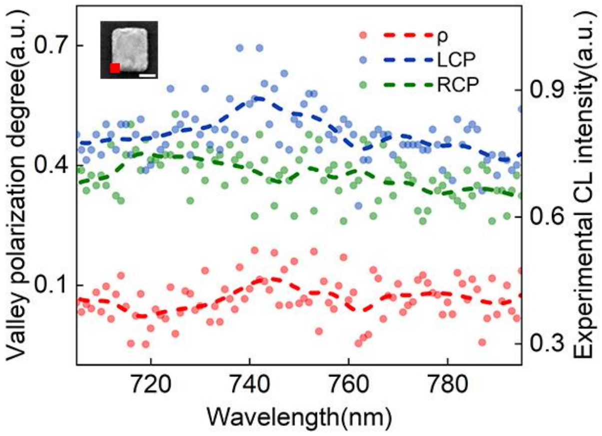

Measured CP-CL spectra and calculated valley polarization degree ρ under electron-beam exciting at bottom-left corner of nanoantenna. The ρ extrated at resonance wavelength is plotted in Figure 3d as the fifth point.

Supplementary Figure 9:

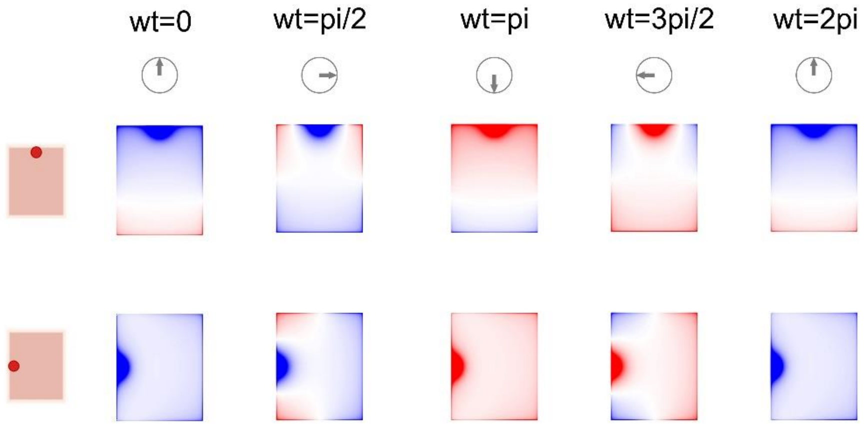

Surface charge distributions with electron beam impinging at top middle and middle left area of Au nanoantenna, showing that these two excitations cannot generate the circular electric dipole.

Supplementary Figure 10:

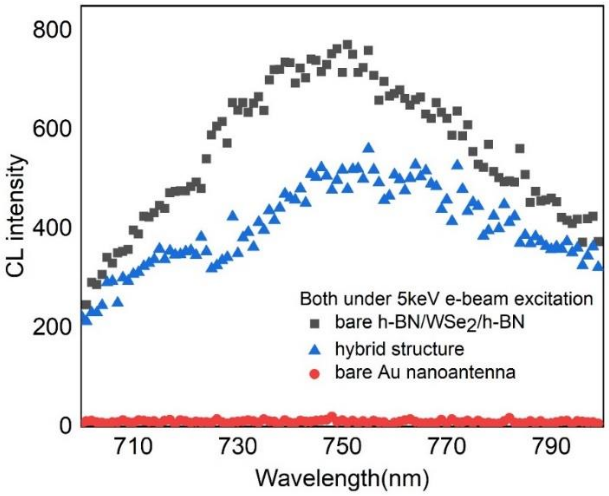

Measured far-field CL intensity that collected from bare Au nanoantenna, bare h-BN/WSe_2/h-BN heterostructure and their hybrid structure. The comparison of these CL spectra directly demonstrates that the CL signals collected with 5 keV, 0.6 nA excitation is dominantly contributed from the WSe_2 monolayer rather than Au.

Supplementary Figure 11:

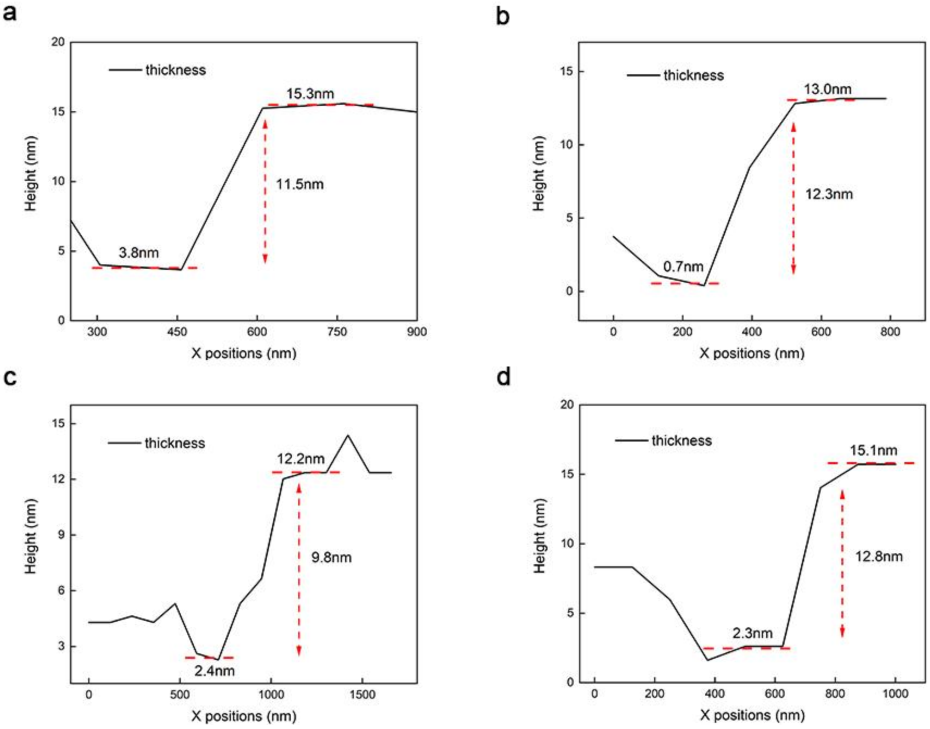

Measured thickness curves of top h-BN in hybrid structure collected at several positions. These results demonstrate that the average thickness of top h-BN is about 10-13nm, showing the homogeneity of top h-BN. Although the top h-BN acts as a spacer between Au nanoantennas and the WSe_2 monolayer, it maybe has few possibilities for occurrence of quenching effect.

Supplementary Figure 12:

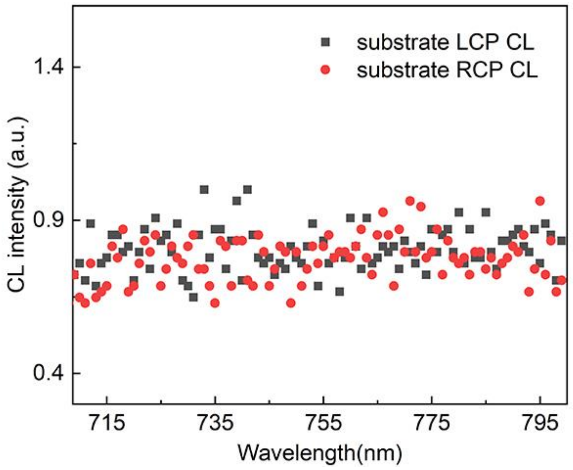

Measured background CP-CL signals of silicon wafer substrate. The almost equivalent LCP and RCP background signals show the calibration of the polarization response.
